# The prolonged devastation of climate change on public health in Somalia: a silent crisis

**DOI:** 10.1186/s41182-025-00856-9

**Published:** 2025-12-25

**Authors:** Saadaq Adan Hussein, Marian Muse Osman, Mohamed Mohamoud Hassan, Yahye Sheikh Abdulle Hassan, Abdirahman Aden Hussein, Rage Adem, Mohamed M. Ali Fuje, Abdinur Hussein Mohamed, Ayan Nur Ali, Khadar Hussein Mohamud, Abdirahman Moallim Ibrahim, AbdulJalil Abdullahi Ali

**Affiliations:** 1 Department of School of Postgraduate Studies, Benadir University, Hodan Benadir, Mogadishu, Somalia; 2 Department of Social and Human Capital Development Pillar, Office of the Prime Minister, Federal Republic of Somalia, Mogadishu, Somalia; 3 Department of Research and Policy Development, SOR Institute: Somalia Social Research, Mogadishu, Somalia; 4 Department Benadir Institute for Research and Development, Benadir University, Mogadishu, Somalia; 5 Department Research, Somali National Institute of Health, Mogadishu, Somalia; 6 Department Office Rector at Benadir University, Mogadishu, Somalia; 7 Department of Medicine and Surgery, Jamhuriya University of Science and Technology, Mogadishu, Somalia; 8 Department Innovation Hub, Benadir University, Mogadishu, Somalia; 9 Department of Emergency, Mogadishu Somali Türkiye Training and Research Hospital, Mogadishu, Somalia; 10 Department Somali Development Research Institute (SODRI), Mogadishu, Somalia; 11 Department Faculty of Medicine and Surgery, Jazeera University, Mogadishu, Somalia

**Keywords:** Climate change, Public health, Scoping review, Somalia, Malnutrition, Infectious diseases, Displacement, Health systems

## Abstract

**Introduction:**

Somalia, one of the world’s most climate-vulnerable nations despite contributing minimally to global emissions, is facing escalating public health crises due to climate change. Rising temperatures, erratic rainfall, recurrent droughts, and floods have intensified food insecurity, disease outbreaks, and population displacement. These changes have compounded existing challenges in a fragile health system, severely affecting children, pregnant women, and internally displaced persons Internally displaced people (IDP). This scoping review aims to map the extent and scope of published evidence on the prolonged public health impacts of climate change in Somalia, identifying key health outcomes, vulnerable populations, and research and policy gaps.

**Methods:**

Following the PRISMA-ScR guidelines, a comprehensive search was conducted in PubMed, Scopus, Web of Science, and Google Scholar, as well as gray literature from WHO, UN agencies, and Somali institutions. Studies published between 1990 and 2025 in English and addressing the intersection of climate change and public health in Somalia or the Horn of Africa were included. Data were charted and synthesized thematically.

**Results:**

Out of 379 retrieved articles, 142 met the inclusion criteria. Key themes emerged: (1) direct health impacts of climate change, (2) indirect health impacts, (3) vulnerable populations, (4) weak health infrastructure and system readiness, and (5) historical droughts and their cumulative health impacts. Children under five, pregnant women, and displaced persons are disproportionately affected. The review highlights significant evidence gaps in mental health, health system resilience, and early warning systems.

**Conclusion:**

This scoping review highlights the severe and multifaceted public health impacts of climate change in Somalia, the fragile healthcare infrastructure in Somalia, and a heavy reliance on external aid. This review provides a foundation for future efforts to mitigate the public health impacts of climate change and build resilience in Somalia and similar vulnerable regions.

## Introduction

Climate change poses a grave threat to global health and human rights by intensifying disease burdens through extreme weather, floods, and the spread of infectious diseases [1–5]. These effects destabilize environmental, agricultural, and economic systems, disproportionately affecting vulnerable populations and sparking humanitarian emergencies [6]. Approximately 90% of climate-related deaths globally are linked to environmental degradation, undernutrition, and infectious diseases, with added psychological trauma [7, 8].

In Africa’s semi-arid regions, including the Horn of Africa, erratic climate patterns worsen food insecurity, displace communities, and deepen poverty across all age groups, with particularly severe impacts on vulnerable populations experiencing climate anxiety [9–11]. Sub-Saharan Africa bears the heaviest burden of malnutrition, with 256.1 million undernourished people—133.1 million in Eastern Africa alone [12, 13]. Somalia, amid political instability and recurring disasters, faces widespread hunger, malnutrition, and health disparities across generations [14–17].

Although contributing just 0.08% to global emissions, Somalia is among the world’s most climate-vulnerable countries [18]. Since 1970, average temperatures have risen by 1.7 °C, accompanied by increasingly severe droughts and floods [19, 20]. Between 2020–2023, Somalia endured five consecutive failed rainy seasons, leading to 70% crop loss, 3 million livestock deaths, and the displacement of 2.9 million people [21–24]. By 2023, 6.6 million faced acute food insecurity and 1.8 million children were at risk of severe malnutrition [25, 26]. El Niño floods in late 2023 further affected 4.3 million people, pushing 1 million into emergency conditions [27, 28].

Climate impacts on the Juba and Shabelle rivers have disrupted agriculture and pastoral livelihoods, threatening the 65% of Somalis reliant on livestock, which contributes 40% to GDP and 80% to foreign earnings [29, 30]. Water scarcity and contamination have fueled outbreaks of cholera, acute watery diarrhea (AWD), and malaria [31–33].while overcrowded camps and low immunization coverage have sustained epidemics of measles, polio, and cholera [34–36]. In 2022, 43,000 excess deaths were recorded half of them children under five [37].

Mental health is also severely affected, with 30% of displaced persons facing depression or anxiety, and one-third of the population experiencing psychological disorders [38–41]. Somalia’s agriculture, dependent on Gu and Deyr seasonal rains, is highly vulnerable due to its arid/semi-arid climate (Köppen B/A) [42–44]. Climate change exacerbates disease transmission and food insecurity, while conflict, underfunding, and weak infrastructure hinder adaptation.

Recent efforts, such as the creation of the Ministry of Environment and Climate Change, are steps forward [45–47]. This comprehensive scoping review approach examines the long-term impacts of climate change, environmental degradation, and public health system breakdown in Somalia, identifying research and policy gaps for resilience-enhancing strategies. This review uses the PRISMA-ScR guidelines to ensure methodological transparency and rigor.

## Material and methods of the study

### Study design and framework

This study employed a scoping review design to explore the prolonged public health impacts of climate change in Somalia. The methodology was guided by the PRISMA-ScR (Preferred Reporting Items for Systematic Reviews and Meta-Analyses extension for Scoping Reviews) checklist, which provides a structured and transparent framework for mapping existing literature on broad and complex topics. Scoping reviews are particularly suited for identifying the nature, range, and gaps in evidence, especially in multidisciplinary fields where heterogeneous sources are used to inform policy and practice. The objective of this scoping review was to systematically identify, categorize, and synthesize the available evidence on how climate change has affected health systems, disease burden, and population vulnerabilities in Somalia from 1990 to 2025, starting from 1990, when the collapse of the central government led to the destruction of health infrastructure and systems, severely impairing the country’s capacity to address climate-related health challenges.

### Eligibility criteria

**Inclusion criteria**

- Publications between January 1990 and March 2025.
- Studies focused on the intersection of climate change and public health in Somalia or the Horn of Africa.
- Peer-reviewed original research, government and NGO reports, systematic or narrative, scoping review. Studies addressing direct (e.g., infectious diseases,) or indirect (e.g., mental health challenges, food insecurity, displacement) public health impacts.

**Exclusion criteria**

- Articles published in languages other than English.
- Studies that focused solely on climate mitigation or environmental science without health-related outcomes.
- Editorials, commentaries, or opinion pieces lacking empirical evidence.

### Search strategy

A comprehensive search was conducted across four major academic databases: PubMed, Scopus, Web of Science, and Google Scholar. In addition, gray literature was sourced from key organizations, including the World Health Organization, United Nations agencies, and relevant reports from Somali government bodies, non-governmental organizations (NGOs) actively working in Somalia, and platforms such as ReliefWeb. These NGOs were selected based on their involvement in health, climate change, and development initiatives within the country, including organizations such as Save the Children, the International Red Cross, Médecins Sans Frontières (MSF), and the Danish Refugee Council. Searches were conducted from December 2024 to February 2025, with final inclusion of studies from March 2025 and limited to English-language publications. The search strategy used a combination of Medical Subject Headings (MeSH) and keywords, adapted for each database. Boolean operators were applied to maximize retrieval: (“climate change” OR “global warming” OR “environmental degradation”) AND (“public health” OR “health outcomes” OR “health systems”) AND (“Somalia” OR “Horn of Africa”) AND (“vector-borne disease*” OR “malnutrition” OR “displacement” OR “mental health impact*” OR “health infrastructure” OR “drought” OR “flood*” OR “climate adaptation*”).

## Study selection and screening process

**Fig. 1:**
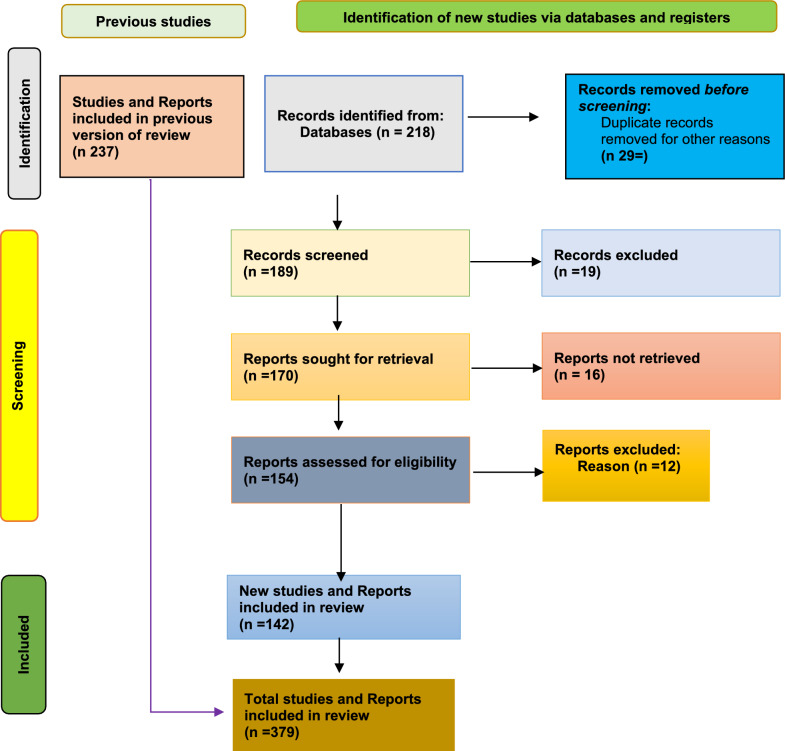
SANRA (scale for the assessment of narrative review articles)(48)

The search retrieved 387 records; after duplicate removal 237 titles and abstracts were screened, yielding141 articles, including qualitative, quantitative, and mixed-methods peer-reviewed studies, were selected for full-text review. Two reviewers independently handled all screening and data extraction, reconciling differences by consensus. Although the study-selection pathway will be depicted in a SANRA (scale for the assessment of narrative review articles) (Fig. 1), no protocol on PROSPERO or OSF.

### Data charting and extraction

A data charting form was developed to extract and organize relevant information from the included studies. Somalia-specific data were identified and extracted where available, and any studies that provided regional data covering the Horn of Africa were carefully reviewed to ensure that Somalia-related findings were distinctly noted. The charting fields included: publication year, study type and setting, health domain (e.g., disease outbreak, malnutrition, mental health), key findings, geographic focus (Somalia or regional) and policy or program implications.

### Data synthesis and thematic analysis

Extracted data were inductively coded in NVivo 12 and, following Braun and Clarke’s six-step thematic analysis, consolidated into five overarching themes:

- Direct health impacts of climate change (e.g., vector- and waterborne diseases, heat-related illness).
- Indirect health impacts (e.g., malnutrition, water scarcity, mental health challenges).
- Vulnerable populations (e.g., children, pregnant women, internally displaced persons).
- Weak health infrastructure and system readiness.
- Historical droughts and their cumulative health impacts.

Findings within each theme were summarized scoping review, drawing attention to gaps, overlaps, and implications for research and policy development in Somalia’s climate-health landscape.

## Result

### Climate change on public health: key impacts

#### Direct health impacts of climate change

##### Increased incidence of vector-borne diseases

Global warming is leading to increased temperatures worldwide, resulting in heat waves and floods [49] that are contributing to the proliferation of vector-borne illnesses such as malaria and dengue fever in Somalia. Malaria remains prevalent in tropical and subtropical regions. Visceral leishmaniasis (VL) has been detected in southern Somalia since 1934, primarily affecting impoverished rural populations and internally displaced individuals. VL continues to be endemic in this area [47, 50]. The 2025 climate forecast predicts reduced rainfall and elevated temperatures in Somalia, increasing the likelihood of expanded malaria transmission areas. Drier conditions are expected in Gedo, Hiraan, and Bay regions, with a 50% chance of below-average rainfall. Above-average temperatures are predicted in Hirshabelle, Galmudug, Puntland, and Somaliland. Significant wet periods will occur from April 20 to May 11 [51].

##### Waterborne diseases

Waterborne diseases significantly burden low-resource countries, particularly in Africa, with broad health and economic impacts [52]. Access to clean water is a fundamental human right, as highlighted by Sustainable Development Goal (SDG) 6, which aims to ensure that everyone has access to affordable drinking water by 2030 [53]. Somalia faces significant challenges in providing its population with safe drinking water. In cities, a little over 76% of households have access to improved water sources. However, this percentage decreases to just above 55% for those living in rural areas and 35% for nomadic communities [54], and internally displaced people (IDP) in Somalia face severe barriers to clean water access, with only 45% of the population using improved sources. Climate-driven droughts, floods, and infrastructure gaps force 22% to rely on unsafe water, heightening disease risk [53]. Major waterborne diseases in Somalia—cholera, typhoid, hepatitis A, and schistosomiasis are driven by unsafe water, poor sanitation, and overcrowding, with frequent cholera and acute watery diarrhea (AWD) outbreaks via fecal–oral transmission [55, 56] and children and elderly individuals are particularly vulnerable to these diseases due to weakened immune systems and poor sanitation infrastructure.

##### Respiratory diseases

The Sahara Desert, known as the world’s largest hot desert, is the primary source of Saharan dust emissions, these dust storms, often referred to as Saharan dust events, mainly occur during the dry season [57]. The presence of Saharan dust has a notable effect on air quality and human health, particularly in areas downwind. It worsens respiratory issues, provokes allergic responses, and heightens cardiovascular risks, especially among vulnerable groups such as children, the elderly, and individuals with existing respiratory conditions [58, 59]. [60, 61]. To strengthen disease surveillance systems, expand vector control programs, and invest in early warning mechanisms for heatwaves and floods to mitigate health risks.

#### Indirect health impacts

##### Food insecurity and malnutrition

Climate change in Somalia is causing food insecurity and malnutrition due to droughts and erratic rainfall, affecting agricultural productivity and livestock, requiring humanitarian assistance for half the population [62]. Somalia has a large agricultural sector, with approximately 70% of the population dependent on agriculture for their livelihood. This includes farmworkers, laborers, and equipment operators. The sector faces frequent climatic shocks, such as droughts, floods, and locust infestations, which contribute to food insecurity and poverty, driving up food prices and inflation [63]. Climate change impacts crop production and livestock husbandry, affecting rural livelihoods and food security. Decreased pasture productivity and animal health, along with livestock diseases, can lead to significant economic losses [63]. Malnutrition weakens immune systems, making individuals more susceptible to infectious diseases, induced immunosuppression leads to heightened morbidity and mortality from infections such as malaria, tuberculosis, and respiratory diseases [64]. Climate-related agricultural shocks disproportionately affect women, youth, and IDP by limiting access to land, resources, and support services, increasing their vulnerability and unemployment [63]. Food security assessments for 2025, Somalians may face crisis levels of food insecurity due to climate shocks, including poor rainfall, crop failures, and livestock losses, as well as disruptions to livelihoods and market access [65].

##### Water scarcity and sanitation issues

In Somalia, 44% of the population lacks basic water services, with many walking over 30 min round-trip to access water. The lack of clean water, sanitation, and hygiene has led to widespread waterborne diseases, disproportionately affecting children and mothers. Ongoing conflict, drought, and food insecurity have also displaced nearly 3 million people, further straining public health systems.[66]. In Somalia, internally displaced people face water scarcity, with only 45% having access to improved sources. Climate shocks and infrastructure gaps leave 22% relying on unsafe water, heightening disease risk [60, 61].

Somalia’s climate crisis has shifted from droughts to 80% water access reduction in rural households and destroying critical infrastructure [67], In Somalia, climate-driven water shortages have reduced access to safe drinking water and sanitation, leading to increased cases of cholera and acute watery diarrhea. The 2020 SHDS revealed widespread use of unimproved water sources, underscoring a critical public health concern [68].

Poor sanitation practices, including open defecation and inadequate infrastructure, increase the spread of waterborne diseases like cholera and typhoid. In Baidoa’s IDP, severe water scarcity forces residents to use contaminated mud for personal hygiene, highlighting the urgent need for safe water access [69]. Recurrent droughts have worsened water access, causing a surge in waterborne diseases. In 2022, Somalia reported over 130,000 cases of acute diarrhea, including 15,600 suspected cholera cases [70]. Efforts such as borehole rehabilitation have improved clean water access, reduced disease prevalence and improving health outcomes [71].

##### Mental health challenges

Climate change has severely impacted mental health in Somalia, particularly among IDP facing displacement and trauma. Studies show 59% exhibit depression symptoms and 32% show signs of PTSD, with women disproportionately affected [72]. According to WHO, 13% of conflict-affected populations suffer from mental health conditions such as depression, anxiety, PTSD, and schizophrenia, with 4% experiencing moderate to severe forms [73].

In Somalia, despite the widespread occurrence of mental health issues, access to mental health services is still quite restricted. Around 80–90% of people experiencing mental health challenges are unable to obtain quality and affordable mental health care. Due to cultural traditions and the scarcity of formal mental health services, many individuals turn to traditional or Koranic healers() for assistance, The Qur’an is the holy book of Islam, believed by Muslims to be the word of God revealed to Prophet Muhammad (peace be upon him) [74]. Climate-driven displacement, conflict, and weak mental health infrastructure highlight the urgent need for integrated, culturally sensitive mental health services within primary care in Somalia. To promote climate-resilient agriculture, improvement in food and water security, and integration of mental health services into humanitarian and primary care responses are needed.

#### Vulnerable populations

##### Children and maternal health

Children and pregnant women in Somalia are highly vulnerable to the health impacts of climate change, particularly due to recurring droughts. As of recent estimates, 1.4 million children under five are acutely malnourished, with over 330,000 at risk of severe acute malnutrition (SAM), affecting nearly half of this age group [75]. Malnutrition increases susceptibility to diseases such as measles, cholera, and pneumonia. In 2022 alone, drought-related conditions led to excess deaths—half among children under five [37].

Pregnant women face similar risks, with Somalia’s maternal mortality ratio at 692 per 100,000 live births—among the highest globally driven by inadequate healthcare and food insecurity [76]. Prolonged droughts are projected to worsen, with forecasts for 2025 indicating that 1.6 million children could be affected by malnutrition, including 403,000 at risk of severe wasting [77].

##### Displaced populations (and refugees)

Globally, 55 million people are internally displaced, with Somalia ranking sixth in total IDP IDP numbers [72, 78, 79]. Over the past 30 years, recurrent droughts and conflict have displaced millions and caused an estimated 256,000 deaths, half among children under five [80]. Most Somali IDP, often from marginalized clans, settle on the outskirts of Mogadishu in overcrowded camps lacking safe water and sanitation [81–83].

These conditions heighten vulnerability to malnutrition, waterborne illnesses, and respiratory infections. Children are twice as likely to suffer from malnutrition and infectious diseases due to food insecurity and limited healthcare access [84]. To Develop targeted interventions for children, pregnant women, and displaced persons through nutrition programs, maternal care, and mobile health outreach.

#### Weak health infrastructure and system readiness

##### Limited health facilities and workforce

Somalia faces a significant healthcare shortage, with only 2.5 physicians and 4.5 nurses per 10,000 people, significantly below the global average [85]. About 30% of the population has access to basic healthcare services, with most in urban areas, making rural and displaced populations vulnerable to health impacts, including climate change-related ones [85, 86]. Rural areas face severe shortages of healthcare facilities, staff, and essential medical supplies, limiting their ability to respond to climate-related health crises like outbreaks and heat-related illnesses [87].

Over 70% of Somalis live below the poverty line, making it difficult for families to afford healthcare services due to high out-of-pocket expenses. This often leads to delayed or missed medical treatments, worsening health outcomes. Somalia’s healthcare financing is characterized by reliance on out-of-pocket payments, further limiting access to services. The lack of public health insurance or free medical care services exacerbates these financial barriers [88].

##### Emergency preparedness and response

Somalia’s emergency preparedness and response systems are ill-equipped to handle the increasing frequency and intensity of climate-related disasters. Somalia faces significant gaps in its disaster response systems, including a lack of robust early warning mechanisms for extreme weather events, inadequate coordination of disaster response efforts, and a shortage of trained personnel and resources [89].

Somalia’s disaster response depends heavily on delayed post-crisis aid, leading to inefficiencies. While financial tools like insurance and trigger-based funding are emerging, they remain underdeveloped. Strengthening early warning systems, coordination among stakeholders, and pre-arranged financing is essential for timely, effective emergency response [90–93]. To increase investment in rural health facilities, workforce training, and emergency preparedness, ensuring equitable healthcare access and climate adaptation capacity.

#### Historical droughts and their cumulative health impacts

##### The Dabadheer (“Long-Tailed”) Drought (1974–1975)

The Dabadheer Drought (1974–1975): triggered by the Sahel drought, this event led to 1,500 deaths and 70–80% livestock losses among northeastern pastoralists. About 250,000 nomads were forcibly resettled, increasing aid dependence [94–98].

##### The 1991–1992 drought and famine

Amid state collapse, drought and famine killed 300,000 people. Crop failures (90% of sorghum/maize) and mass displacement marked the beginning of Somalia’s failed state period [98–101].

##### III. The 2010–2012 Horn of Africa drought

La Niña-related rainfall failure and conflict led to 133,000 child deaths and 1.5 million displacements. Estimated $1.2 billion in agricultural losses occurred [102–105].

##### The 2015–2016 El Niño drought

This El Niño event caused four failed seasons in Puntland and Somaliland, affecting 1.4 million people. Livestock deaths and food insecurity worsened economic hardship [102, 106, 107].

##### The 2016–2017 back-to-back droughts

Two consecutive failed rainy seasons left 6.2 million in need, with 1.4 million children malnourished and 60% livestock lost in the north, driving rural-to-urban migration [108, 109].

##### The 2020–2023 historic drought

Five failed seasons caused deaths in 2022 (half were children), displaced 1.3 million, and led to the worst malnutrition crisis since 2011, affecting 8.3 million people [37].

##### The 2024 drought crisis

From Jan 2022 to June 2024, an ongoing drought caused 71,100 excess deaths—40% among children under five—highlighting worsening mortality and displacement [37]. To implement long-term drought mitigation strategies, enhance community resilience through water conservation, and integrate lessons learned into national climate-health policies.

## Discussion

The worsening climate crisis in Somalia has resulted in an extraordinary humanitarian and public health emergency. This scoping review provides an integrated overview of the multi-dimensional impacts of climate change on Somalia’s public health landscape. By synthesizing diverse sources, it highlights both well-documented areas (e.g., food insecurity) and underexplored domains (e.g., mental health, early warning systems). Unlike systematic reviews, the goal here was not to evaluate effect size but to map the breadth and gaps in knowledge to inform future strategies.

Climate change is an escalating global crisis, with severe effects in fragile states like Somalia. Despite contributing only 0.08% to global greenhouse gas emissions [110], Somalia is highly vulnerable to climate-related shocks. The increasing frequency and severity of droughts, floods, and extreme weather events have worsened food insecurity, malnutrition, disease outbreaks, and mental health disorders, creating a silent crisis affecting Somalia’s most vulnerable populations, including children, women, and IDP.

Somalia is among the countries most severely impacted by climate change, a phenomenon also evident in various regions across Africa and Asia. The Sahel region parallel, for instance, frequently experiences droughts, desertification, and food shortages, leading to displacement and health crises [111]. Similarly, Bangladesh contrast, faces climate-induced flooding, which exacerbates the spread of diseases such as cholera and dengue fever [112]. However, unlike these countries, Somalia’s prolonged conflict and inadequate healthcare infrastructure exacerbate its climate vulnerabilities, complicating efforts to adapt and mitigate these challenges.

Analyzing past drought occurrences, such as the 1974–1975 “Dabadheer” drought, the 1991–1992 famine, and the 2010–2012 crisis, reveals that Somalia has consistently been prone to climate-induced challenges [94]. However, the recent droughts from 2020 to 2023 highlight a worrisome escalation in severity and impact. Unlike the earlier droughts, which lasted one or two years, the recent drought extended over five seasons (2020–2023), marking it as the longest and most intense in Somalia’s history [113]. This prolonged drought led to famine-like conditions, resulting in the displacement of 1.3 million people and the deaths of approximately individuals in 2022 alone, with half of the deceased being children under the age of five [37].

Somalia is facing an ongoing food insecurity crisis, which has been intensified by climate change, resulting in persistent malnutrition among children and pregnant women. When comparing the famine of 2011 to the crisis in 2022, it is evident that although there have been advancements in humanitarian response efforts, the severity of food insecurity remains a significant concern [114, 115]. In 2011, the UN declared a famine after 260,000 individuals lost their lives, primarily due to limited access for humanitarian aid in regions controlled by insurgents. Conversely, in 2022, Somalia encountered its most severe malnutrition crisis in a decade, with 1.8 million children experiencing acute malnutrition, despite improved early warning systems and relief initiatives [105]. Unlike previous famines, which were mainly caused by conflict-related food shortages, the drought from 2020 to 2023 severely damaged Somalia’s agricultural sector, leading to crop failures, livestock deaths, and a collapse in food production. Climate predictions for 2025 indicate that 4.4 million people could face crisis-level food insecurity, further worsening an already dire situation, those grappling with a severe drought, causing 70% crop destruction and 3 million livestock deaths, marking the worst drought in 40 years [65].

By 2023, 6.6 million people in Somalia were experiencing acute food insecurity, with 1.8 million children at risk of severe malnutrition [25]. In comparison, Ethiopia, which has a more robust agricultural infrastructure, has managed to somewhat alleviate food crises through government-led climate resilience initiatives. Conversely, Somalia’s dependence on subsistence farming and pastoralism, along with issues of insecurity and poor governance, has rendered long-term food security efforts largely ineffective [116, 117].

Climate change has exacerbated the spread of vector-borne diseases like malaria, dengue fever, and visceral leishmaniasis. The increase in temperatures and extended wet seasons have enlarged mosquito breeding areas, leading to a rise in malaria transmission, especially in southern Somalia [118, 119]. This pattern is similar to what is observed in West Africa parallel, where malaria. Furthermore, frequent floods and droughts have triggered significant outbreaks of waterborne diseases, such as cholera, acute watery diarrhea (AWD), and hepatitis A. The floods caused by the 2023 El Niño event impacted 4.3 million individuals, polluting water supplies and deteriorating sanitation conditions, Comparable issues are seen in Pakistan, where climate-driven flooding has resulted in cholera and typhoid fever outbreaks, straining health systems [120, 121].

Somalia faces a severe food insecurity crisis, exacerbated by climate change, causing malnutrition among children and expectant mothers. Comparing the 2011 famine with the 2022 crisis shows that despite improved humanitarian responses, food insecurity remains deeply concerning [122]. In 2011, the UN declared a famine after 260,000 people died, mainly due to restricted humanitarian access in insurgent-controlled areas. In 2022, Somalia faced its worst malnutrition crisis in a decade, with 1.8 million children suffering acute malnutrition, despite improved early warning systems and relief efforts [123]. Unlike earlier famines driven by conflict-related food insecurity, the 2020–2023 drought severely impacted Somalia's agricultural sector, causing crop failures, livestock deaths, and collapsed food production. Climate forecasts for 2025 predict many people could face crisis-level food insecurity, worsening the critical situation [65].

The link between climate change and vector-borne diseases in Somalia is becoming more apparent. Rising temperatures and erratic rainfall have broadened the areas susceptible to malaria transmission, especially in regions where the disease was once uncommon, the climate projections for 2025 indicate a decline in rainfall and an increase in temperatures in areas like Gedo, Hiraan, and Bay, which could heighten the risk of malaria and outbreaks[31, 124]. Likewise, waterborne diseases, notably cholera and acute watery diarrhea (AWD), have surged in IDP, settlements and regions prone to flooding. In 2022, there were over 130,000 cases of acute diarrheal diseases, including 15,600 suspected cholera cases, marking the highest number in five years [125]. A review of past outbreaks reveals that flooding is increasingly significant in disease outbreaks, as demonstrated by the El Niño-induced floods of 2023, which displaced 4.3 million people and tainted water supplies.

Climate change has exacerbated the spread of vector-borne diseases like malaria, dengue fever, and visceral leishmaniasis [118, 126]. The increase in temperatures and extended wet seasons have enlarged mosquito breeding areas, leading to a rise in malaria transmission, especially in southern Somalia [123]. This pattern is similar to what has been observed in West Africa, where malaria cases have surged due to higher temperatures [124, 127, 128]. Furthermore, frequent floods and droughts have triggered significant outbreaks of waterborne diseases, such as cholera, acute watery diarrhea (AWD), and hepatitis A. The floods caused by the 2023 El Niño event impacted 4.3 million individuals, polluting water supplies and deteriorating sanitation conditions. Comparable issues are seen in Pakistan, where climate-driven flooding has resulted in cholera and typhoid fever outbreaks, straining health systems [129, 130].

Climate change has not only impacted physical health, but has also intensified mental health challenges, particularly for internally displaced persons IDP and communities affected by conflict. According to research, 30% of displaced Somalis are dealing with depression or anxiety [131], and 13% are experiencing post-traumatic stress disorder (PTSD). This issue extends beyond Somalia. In the Philippines, where typhoons and extreme weather are frequent, climate-related anxiety is becoming a significant concern, especially among the youth [132]. While the Philippines is working to integrate mental health services into primary healthcare, Somalia faces a shortage of psychiatric care, leading many to turn to traditional healing methods [133].

Climate change has significant psychological and emotional impacts, especially on internally displaced persons IDP and vulnerable groups. In past crises, like the famine of 1991–1992, mental health issues were often overlooked as the focus was on immediate survival. However, recent research shows that displacement due to climate change has resulted in increased cases of depression, anxiety, and post-traumatic stress disorder (PTSD) among those affected [134, 135]. In Somalia, the mental health toll is severe. A 2023 study in Mogadishu found that 59% of IDP exhibited symptoms of depression, while 32% met the criteria for PTSD [134]. Unlike in previous decades, when mental health services were nearly absent, there have been recent attempts to incorporate mental health into primary healthcare systems. Nevertheless, Somalia still faces a shortage of mental health professionals, with only 0.5 psychiatrists available per 100,000 people, severely limiting access to care [136].

Somalia’s healthcare system is too fragile to address the growing diseases caused by climate change. With a severe shortage of doctors and limited medical facilities in rural areas, healthcare access remains among the lowest in the world [137]. The high out-of-pocket expenses for healthcare further hinder marginalized groups from obtaining necessary treatment, resulting in increased illness and death rates [137]. In contrast, nations like Kenya and Rwanda have heavily invested in universal health coverage (UHC) and climate-resilient health systems, leading to better health outcomes even in areas vulnerable to climate change [138]. To enhance resilience, Somalia urgently needs to invest in primary healthcare infrastructure, early warning systems, and climate adaptation strategies.

Over the last 20 years, climate-related displacement in Somalia has significantly increased, more than 3.8 million individuals were forced to move due to climate-related disasters, positioning Somalia as one of the most impacted nations worldwide [139, 140]. The drought from 2020 to 2023 alone displaced 1.3 million people, a number similar to the displacement caused by conflicts in the 1990s. Displacement leads to numerous public health issues, especially in IDP, settlements, where overcrowding, poor sanitation, and restricted access to healthcare result in adverse health outcomes. Unlike in past decades, when IDP, settlements were mostly temporary, many displaced communities now live in semi-permanent, urbanized areas, leading to enduring public health challenges. Additionally, Somalia's fragile healthcare system continues to hinder climate adaptation efforts. The nation suffers from a severe shortage of physicians and nurses, well below the global average [85]. This deficiency hampers Somalia's capacity to handle climate-related health emergencies, as demonstrated by the 2022 cholera outbreak, where limited healthcare services worsened mortality rates. This review is limited by its exclusion of non-English literature and potential bias in gray literature. Additionally, while efforts were made to chart evidence comprehensively, some recent unpublished studies may have been missed.

## Limitation

This scoping review has several limitations. First, a formal protocol was not registered (e.g., on OSF or PROSPERO), which is acknowledged as a methodological constraint. The reliance on gray literature, including reports from WHO, UN agencies, and Somali institutions, introduces potential biases related to reliability, publication bias, and lack of peer review. Although efforts were made to triangulate findings from multiple sources to enhance credibility, the absence of peer-reviewed sources may limit the overall rigor of the evidence. Additionally, the review excluded studies published in languages other than English, potentially restricting the breadth of evidence, particularly from Somali-language sources that could offer valuable local insights. Finally, some unpublished studies were not included, and their exclusion may have resulted in missed critical data. The fragility of Somalia’s healthcare infrastructure also presents challenges, as the data on health system resilience may not fully reflect the capacity or preparedness to address climate-induced health crises.

## Conclusion

This scoping review highlights the severe and multifaceted public health impacts of climate change in Somalia. The country, despite contributing minimally to global emissions, remains highly vulnerable to climate-related crises, including extreme weather events, recurrent droughts, and floods. These climatic changes exacerbate existing health challenges, contributing to increased food insecurity, malnutrition, disease outbreaks, and mental health issues, particularly among vulnerable populations such as children, pregnant women, and internally displaced persons (IDPs).

The review identified significant evidence gaps, particularly in mental health, health system resilience, and early warning systems. The fragile healthcare infrastructure in Somalia, coupled with inadequate resources and a heavy reliance on external aid, hampers the country's ability to effectively respond to climate-induced health emergencies. Despite some progress, such as the establishment of the Ministry of Environment and Climate Change, Somalia’s healthcare system remains ill-equipped to handle the escalating health threats posed by climate change.

The findings underscore the urgent need for multisectoral action to strengthen climate-resilient health systems, enhance early warning capacities, and prioritize the health needs of vulnerable populations. Further research and policy development are crucial to addressing these challenges and improving health outcomes in Somalia’s fragile context. This review provides a foundation for future efforts to mitigate the public health impacts of climate change and build resilience in Somalia and similar vulnerable regions.

## Data Availability

Our correspondents are available to provide raw data at any time, ensuring the smooth functioning of our initiatives.

## Declarations

### Descriptions for experiments

No experiments involving human or animal subjects were conducted. This study is based on a scoping review of published literature and publicly available data sources.
